# Pharmacologic inhibition of S-nitrosoglutathione reductase protects against experimental asthma in BALB/c mice through attenuation of both bronchoconstriction and inflammation

**DOI:** 10.1186/1471-2466-14-3

**Published:** 2014-01-10

**Authors:** Joan P Blonder, Sarah C Mutka, Xicheng Sun, Jian Qiu, Lucia H Green, Navdeep K Mehra, Ramakrishna Boyanapalli, Michael Suniga, Kirsten Look, Chris Delany, Jane P Richards, Doug Looker, Charles Scoggin, Gary J Rosenthal

**Affiliations:** 1 N30 Pharmaceuticals, Inc, 3122 Sterling Circle, Suite 200, Boulder, CO 80301, USA

**Keywords:** Asthma, Inflammation, Mouse, Ovalbumin, S-nitrosoglutathione reductase, S-nitrosoglutathione, Nitric oxide, N6022, NFκB

## Abstract

**Background:**

S-nitrosoglutathione (GSNO) serves as a reservoir for nitric oxide (NO) and thus is a key homeostatic regulator of airway smooth muscle tone and inflammation. Decreased levels of GSNO in the lungs of asthmatics have been attributed to increased GSNO catabolism via GSNO reductase (GSNOR) leading to loss of GSNO- and NO- mediated bronchodilatory and anti-inflammatory actions. GSNOR inhibition with the novel small molecule, N6022, was explored as a therapeutic approach in an experimental model of asthma.

**Methods:**

Female BALB/c mice were sensitized and subsequently challenged with ovalbumin (OVA). Efficacy was determined by measuring both airway hyper-responsiveness (AHR) upon methacholine (MCh) challenge using whole body plethysmography and pulmonary eosinophilia by quantifying the numbers of these cells in the bronchoalveolar lavage fluid (BALF). Several other potential biomarkers of GSNOR inhibition were measured including levels of nitrite, cyclic guanosine monophosphate (cGMP), and inflammatory cytokines, as well as DNA binding activity of nuclear factor kappa B (NFκB). The dose response, onset of action, and duration of action of a single intravenous dose of N6022 given from 30 min to 48 h prior to MCh challenge were determined and compared to effects in mice not sensitized to OVA. The direct effect of N6022 on airway smooth muscle tone also was assessed in isolated rat tracheal rings.

**Results:**

N6022 attenuated AHR (ED_50_ of 0.015 ± 0.002 mg/kg; Mean ± SEM) and eosinophilia. Effects were observed from 30 min to 48 h after treatment and were comparable to those achieved with three inhaled doses of ipratropium plus albuterol used as the positive control. N6022 increased BALF nitrite and plasma cGMP, while restoring BALF and plasma inflammatory markers toward baseline values. N6022 treatment also attenuated the OVA-induced increase in NFκB activation. In rat tracheal rings, N6022 decreased contractile responses to MCh.

**Conclusions:**

The significant bronchodilatory and anti-inflammatory actions of N6022 in the airways are consistent with restoration of GSNO levels through GSNOR inhibition. GSNOR inhibition may offer a therapeutic approach for the treatment of asthma and other inflammatory lung diseases. N6022 is currently being evaluated in clinical trials for the treatment of inflammatory lung disease.

## Background

S-nitrosoglutathione (GSNO) is formed through the reaction of glutathione with reactive nitrogen species and serves as the main reservoir of cellular S-nitrosothiol (SNO) species that govern total and/or local nitric oxide (NO) bioavailability *in vivo*[1,2]. GSNO and SNOs serve as functional depots for NO [2,3] which has a short biological half-life [4]. Increases in bio-available NO are associated with anti-inflammatory and smooth muscle relaxant effects, especially in organ systems characterized by smooth muscle and endothelial/epithelial layers such as the respiratory, cardiovascular, and gastrointestinal systems [5,6]. In particular, NO and GSNO help to maintain normal lung physiology and function via the actions of these mediators on bronchial smooth muscle tone and responsivity, adrenergic receptor function, and anti-inflammatory activities [7-9].

GSNO is catabolized by S-nitrosoglutathione reductase (GSNOR), a class III alcohol dehydrogenase (ADH) [10,11]. Therefore, GSNOR has an important role in regulating intracellular SNOs and, subsequently, the function of these compounds [11], while dysregulation of this enzyme can lead to deleterious effects as observed in respiratory and other diseases [12,13]. Specifically, there are lowered SNO concentrations in the lungs of asthmatic patients which have been attributed to up-regulated GSNOR activity [13,14]. Furthermore, various alleles of the human GSNOR gene have been associated with an increased risk of childhood asthma and with a decreased response to albuterol among different ethnic populations [15-17]. The increased GSNOR activity with subsequent loss of GSNO, SNOs, and their associated activities, points to this enzyme as a potential therapeutic target especially in the treatment of respiratory diseases including asthma.

In fact, there is both preclinical and clinical evidence supporting a role for inhibiting GSNOR in the treatment of asthma. Que *et al.* (2005) showed that mice with genetic deletion of GSNOR were protected from methacholine (MCh)-induced airway hyper-responsiveness (AHR) following ovalbumin (OVA) sensitization and challenge [18]. SNOs were found to be lowered in tracheal irrigations in asthmatic children with respiratory failure in comparison to normal children undergoing elective surgery [14]. SNO content was decreased in the bronchoalveolar lavage fluid (BALF) in adult patients with mild asthma compared to healthy control subjects, and was inversely correlated with GSNOR expression in BALF cell lysates [13]. Furthermore, GSNOR activity in BALF cell lysates was significantly increased in asthmatics compared to controls and correlated with increased MCh responsivity [13].

Exhaled NO is increased in patients with severe asthma [19,20] and the lowering of this parameter is used as a measure of the anti-inflammatory effectiveness of therapeutics [21]. The increased NO in asthma has been attributed to generation from inducible nitric oxide synthase (iNOS) in response to inflammatory signals typical in this disease, and NO generated in this manner can have pro-inflammatory activity [20]. Inhibitors of iNOS have been developed for the treatment of respiratory diseases, including asthma, in attempts to mitigate the NO mediated inflammatory signals [22,23]. Conversely, NO donors have also been developed for the treatment of respiratory diseases for their bronchodilatory and anti-inflammatory benefits [24,25]. These contradictions surrounding NO may be attributable to the source (*i.e*., NOS isoform), amount, and location of NO production as well as pathways involved in NO processing, signaling, or metabolism [19,26].

As evident in asthma, increased GSNOR activity leads to lowered GSNO and SNOs [13] in spite of the increased NO. Similar conditions with increased NO and inflammation, but potentially lowered SNOs and decreased SNO-mediated function, are evident in non-respiratory diseases, including cardiovascular disease [27,28] and inflammatory bowel disease [29], in which a role for GSNOR may exist [27,30]. GSNOR dysregulation may therefore help explain the decreased pool of bioavailable NO in disease states in the presence of a pro-inflammatory NO signal.

This study evaluated the potential of GSNOR inhibition as a therapeutic approach in the treatment of asthma. Specifically, the effects of N6022, a novel, potent, and selective small molecule inhibitor of GSNOR [31,32], were evaluated in a murine model of asthma induced by systemic sensitization followed by airway challenges with OVA. Endpoints measured were AHR in response to aerosol challenge with MCh using non-invasive plethysmography [33] as well as eosinophilic infiltration into the BALF. Other determinations included assessments of nitrite, cyclic guanosine monophosphate (cGMP), and biomarker profiles in plasma and BALF, nuclear factor kappa B (NFκB) activity in the lung, and modulation of airway smooth muscle tone in a tracheal ring bioassay. These studies showed that inhibition of GSNOR activity with a single intravenous (i.v.) dose of N6022 imparted potent effects against key parameters in asthma, specifically, AHR and eosinophilic inflammation, with mechanisms consistent with restoring normal levels and function of SNOs in the airways. N6022 is currently being evaluated for safety and efficacy in clinical trials based on these findings and the role of GSNOR in disease.

## Methods

### Drug information

N6022, 3-(5-(4-(1H-imidazol-1-yl) phenyl)-1-(4-carbamoyl-2-methylphenyl)-1H-pyrrol-2-yl) propanoic acid, was synthesized at N30 Pharmaceuticals, Inc. [32]. N6022 has been shown to be a potent, selective, and reversible inhibitor for human GSNOR [31,32]. N6022 also has been shown to be well tolerated in animals [34].

### Animals

The mouse OVA study protocol was approved by the Institutional Animal Care and Use Committee and attending veterinarian at BioTox Sciences, Inc. (San Diego, CA) following guidelines provided and required under the United States Department of Agriculture (USDA) Animal Welfare Act (AWA) and with approval from the Office of Laboratory Animal Welfare (OLAW). Female BALB/c mice, 6 to 9 weeks of age at study initiation, were obtained from Harlan (Indianapolis, IN) and housed at BioTox Sciences. The in-life portion of the OVA studies were performed at BioTox Sciences with additional analyses conducted on study samples at N30 Pharmaceuticals, Inc. (Boulder, CO).

The rat tracheal ring protocol was approved by the IACUC and attending veterinarian at Bolder BioPATH, Inc. (Boulder, CO) following the USDA-AWA and OLAW guidelines and approval. For tracheal ring bioassays, male Sprague Dawley rats that were 8 to 10 weeks of age and weighing 250 to 300 g were obtained from Harlan (Indianapolis, IN) and housed at Bolder BioPATH. Tissues were harvested at Bolder BioPATH with additional processing and bioassay conducted at N30 Pharmaceuticals.

### Drug administration

N6022 was reconstituted in Ca^2+^- and Mg^2+^-free phosphate buffered saline (PBS), pH 7.4, and administered to mice as a single i.v. dose. In the dose response studies, N6022 doses ranging from 0.001 mg/kg to 30 mg/kg were given 24 h prior to the MCh challenge. PBS vehicle was used as a control and was given as a single i.v. administration 24 h prior to MCh. In the time course studies, N6022 was administered i.v. at 0.1 mg/kg or 10 mg/kg from 1 h to 48 h or from 30 min to 8 h prior to the MCh challenge. PBS vehicle was administered at either 24 h or 8 h in these studies. A combination of ipratropium bromide and albuterol sulfate (IpBr + Alb.; Combivent®, Boehringer) was used as the positive control for all studies. IpBr + Alb was delivered to the lung via inhalation (IH) as three doses, one dose each at 48 h, 24 h, and 1 h prior to MCh challenge. Each dose delivered 0.02 mg (0.9 mg/kg) IpBr and 0.1 mg (5.2 mg/kg) Alb for a total dose of 2.7 mg/kg IpBr and 15.6 mg/kg Alb. Administration of N6022, IpBr + Alb, and PBS at 24 h prior to MCh challenge occurred on the same day as the last OVA airway challenge which was given on study day 22 (see below). In these instances, compounds were administered one hour prior to OVA.

### OVA sensitization

OVA was dissolved in PBS at 0.5 mg/mL and aluminum potassium sulfate (alum) was prepared at 10% (w/v) in distilled water. Equal volumes of both solutions were mixed together, the pH was adjusted to 6.5 using 10 N NaOH, and the mixture was incubated for 60 min at room temperature. This mixture was centrifuged at 750 × g for 5 min and the OVA/alum pellet was resuspended in distilled water. Mice received an intraperitoneal (i.p.) injection of 100 μg OVA complexed with 20 mg alum in a volume of 0.2 mL on study day 1. For OVA airway challenges, mice were anesthetized with an i.p. injection of 0.44 mg/kg ketamine and 6.3 mg/kg xylazine in 0.2 mL volume and placed on a board in the supine position. OVA solution was applied intra-tracheally on days 9, 16, 19, and 22. Mice received 250 μg OVA in 0.1 mL on day 9, and 125 μg OVA in 0.05 mL on days 16, 19, and 22.

### AHR measurement

*In vivo* airway responsiveness to MCh was measured in conscious, unrestrained, spontaneously breathing mice with whole body plethysmography using a Buxco chamber (Wilmington, NC). Baseline measurements were obtained, and mice were then challenged with aerosolized saline, followed by increasing doses of MCh (5, 20, and 50 mg/mL) generated by an ultrasonic nebulizer. MCh exposure times were five min with a one min recovery between subsequent doses. The degree of AHR was expressed as enhanced pause (Penh) which correlates with the measurement of airway resistance, impedance, and intrapleural pressure. Penh readings were averaged over 4 min after each nebulization challenge. Penh was calculated as follows: Penh = [(T_e_/T_r_ – 1) × (PEF/PIF)], where T_e_ was expiration time, T_r_ was relaxation time, PEF was peak expiratory flow, and PIF was peak inspiratory flow × 0.67 coefficient. The time for the box pressure to change from a maximum to a user-defined percentage of the maximum represented the relaxation time. The T_r_ measurement began at the maximum box pressure and ended at 40%.

### Pulmonary inflammation

After measurement of AHR, the mice were euthanized and BALF was collected from the right lung after tying off the left lung at the mainstem bronchus. The right lung was lavaged three times with 0.4 mL PBS per wash. In some studies, BALF was collected from both lungs by lavaging four times with 1 mL PBS per wash. Total BALF cell numbers were counted with a hemacytometer, the fluid was centrifuged at 200 × g for 10 min at 4°C, and a Cytospin slide of resuspended cells was prepared. Eosinophils were quantified via light microscopy using Diff-Quik stain (Dade Behring) and morphological criteria. Eosinophil percent was expressed as percent of total BALF cells and as percent relative to the vehicle control in each study.

### Tissue collection

Blood was collected into K_2_EDTA tubes and plasma was obtained via centrifugation. Plasma, lungs, and BALF supernatant (above) were snap frozen in liquid nitrogen and stored at −80°C until analyzed.

### Biomarker profiles in BALF and plasma

Inflammatory biomarker patterns in BALF and plasma were assessed in a multi-analyte panel via immunoassay (Rodent MAP®v2.0, Myriad-RBM, Austin, TX). Additional plasma biomarkers included measurement of matrix metalloproteinase 9 (MMP-9) using the Quantikine® Mouse MMP-9 (total) Immunoassay (R&D Systems, Minneapolis, MN), RANTES (regulated upon activation normal T-cell expressed and secreted) using the Quantikine® Mouse RANTES Immunoassay (R&D Systems), and cGMP using the colorimetric Enzyme Immunoassay Direct cGMP kit (Sigma). MMP-9, RANTES, and cGMP assays were performed according to instructions provided by the manufacturer with detection using a SpectraMax M2 plate reader (Molecular Devices, Sunnyvale, CA).

### NFκB functional assay

All supplies were obtained from Thermo Scientific (Rockford, IL). Nuclear proteins were extracted from lung homogenates using NE-PER Nuclear and Cytoplasmic Extraction reagents following the supplied procedures. Protein concentration in the nuclear fractions was determined via the bicinchoninic acid method. The binding of the NFκB p65 subunit to NFκB consensus sequence DNA was assessed as an index of NFκB function using the NFκB p65 Transcription Factor Assay Kit and the supplied procedures.

### Nitrite determinations

BALF nitrite was measured using ozone chemiluminescence detection (Sievers Nitric Oxide Analyzer, Boulder, CO) following tri-iodide reduction of nitrite to nitric oxide [35].

### Tracheal ring bioassay

Tracheal rings containing 3 to 4 cartilage rings were mounted at isometric tension in a small vessel wire myograph (DMT 610 M, DMT-USA, Atlanta, GA) in Krebs bicarbonate buffer containing 119 mM NaCl, 4.7 mM KCl, 1.2 mM MgSO_4_, 1.2 mM KH_2_PO_4_, 2.5 mM CaCl_2_, 25 mM NaHCO_3_, 0.03 mM EDTA, and 5.5 mM glucose and continuously gassed with 95% O_2_: 5% CO_2_. Tracheal rings were equilibrated at a resting tension of 1 g for one hour and then treated with 100 μM N6022, 100 μM albuterol, or PBS vehicle for 30 min. MCh was added in cumulative doses ranging from 0.01 μM to 100 μM to induce smooth muscle contraction. In other assays, tracheal rings were contracted with 1 μM MCh (effective concentration 40%, EC_40_) and then treated with 0.3 to 100 μM N6022 or GSNO to induce relaxation. Control rings were treated with equivalent volumes of PBS vehicle. Data were acquired and analyzed using Powerlab (ADInstruments, Colorado Springs, CO). Additional data analyses were performed in GraphPad Prism 5.0 (La Jolla, CA). The amount of contraction was reported as the percent of maximum contraction achieved in vehicle control. The amount of relaxation was reported as the percent of possible maximum relaxation achievable per ring, i.e., peak MCh response minus the resting tension.

### Statistical analyses

All data are presented as means ± SEM. Statistical analyses for Penh, eosinophils, and biomarkers were performed using a One-way ANOVA followed by Dunnett’s post-hoc test or a two-tailed Student’s t-test using JMP 8.0 software (SAS Institute, Cary, NC). Statistical analyses for the tracheal ring bioassay were performed using a Two-way ANOVA with treatment and dose as variables, followed by Bonferroni’s post-hoc test (GraphPad Prism). Differences between treatment and control groups were considered significant at p < 0.05. The dose of N6022 that decreased Penh by 50% (ED_50_) was calculated at 5, 20, and 50 mg/mL MCh using GraphPad Prism.

## Results

### N6022 dose response studies

**Figure 1:**
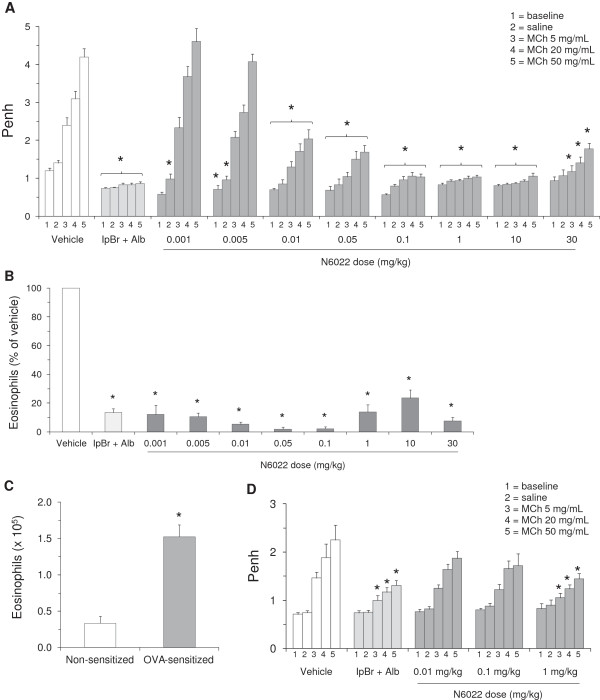
**N6022 caused a dose-dependent decrease in Penh and BALF eosinophils in OVA-sensitized and non-sensitized mice.** Non-sensitized or OVA-sensitized mice were treated with N6022 or PBS vehicle given via a single i.v. dose at 24 h prior to MCh challenge. IpBr (0.9 mg/kg/dose) + Alb (5.2 mg/kg/dose) was administered as inhaled doses at 48 h, 24 h, and 1 h prior to MCh as a positive control for the model. Dosing at 24 h prior to MCh challenge occurred one hour prior to the last OVA challenge on study day 22. Penh was measured at baseline and after challenge with saline followed by increasing doses of MCh aerosol. BALF eosinophils were quantified via light microscopy. N6022 attenuated Penh **(A)** and BALF eosinophils **(B)** in OVA-sensitized mice. OVA sensitization significantly increased BALF eosinophils **(C)** and Penh (**D** vs. **A**, Vehicle). N6022 attenuated Penh in non-sensitized mice **(D)**. Bars are the means ± SEM of 10 to 30 mice per group for **(A)**, **(B)**, and **(C)** and 10 mice per group for **(D)**. *p < 0.05 for treatment vs. vehicle, One-way ANOVA, Dunnett’s for **(A)**, **(B)**, and **(D)**. *p < 0.05 for OVA-sensitized vs. non-sensitized, two-tailed Student’s t-test for **(C)**.

The GSNOR inhibitor, N6022, demonstrated potent effects in a mouse model of OVA-induced asthma. When administered as a single i.v. dose at 24 h prior to MCh challenge, N6022 caused a significant and dose-dependent attenuation of Penh upon challenge of mice with increasing doses of MCh aerosol (Figure 1A). Significant attenuation of the MCh induced increases in Penh was evident at doses of N6022 ranging from 0.01 mg/kg to 30 mg/kg when compared to vehicle treated mice. N6022 at doses ≥ 0.005 mg/kg also caused a significant lowering of Penh values measured at baseline and upon exposure to saline aerosol compared to vehicle treated mice (Figure 1A). The ED_50_ for N6022 from these studies was determined to be 0.015 ± 0.002 mg/kg.

N6022 also decreased the percent of BALF eosinophils (Figure 1B), which were significantly elevated in the OVA model as expected (Figure 1C). Significant lowering of eosinophils was achieved at all doses (0.001 to 30 mg/kg) of N6022 when compared to vehicle treated mice.

The bronchodilatory and anti-inflammatory actions of N6022 in the OVA mice were evident after administration of a single i.v. dose given after three of the four airway challenges with OVA, and 24 h prior to the MCh challenge. In addition, the effects of N6022 were similar to those observed for the positive control utilized for this model, a combination of IpBr + albuterol, which was administered as three inhaled doses prior to the MCh challenge (Figures 1A and 1B).

The action of N6022 was tested in mice that were not sensitized to OVA in order to determine the ability of this compound to directly influence airway smooth muscle tone in the absence of ongoing inflammatory processes. N6022 significantly attenuated the increase in Penh upon exposure to all doses of MCh aerosol when administered as a single 1 mg/kg i.v. dose which was the highest dose tested in this study (Figure 1D). As observed in the OVA model, this effect of N6022 was similar to the effect of three inhaled doses of IpBr + Alb.

### Biomarker profiles

When biomarker patterns were profiled in BALF collected from the OVA dose response studies, several biomarkers associated with asthma and inflammation were significantly elevated upon OVA exposure (Table 1). In particular, eotaxin, several interleukins (IL) including IL-4 and IL-5, inflammatory cell chemoattractants including keratinocyte chemoattractant/growth related oncogene alpha (KC/GROα), macrophage inflammatory proteins (MIPs), monocyte chemotactic proteins (MCPs), and RANTES, and other mediators released from inflammatory cell infiltrates including MMP-9, myeloperoxidase (MPO), and tumor necrosis factor alpha (TNFα) were either significantly increased or achieved detectable levels in OVA-sensitized mice compared to non-sensitized mice (Table 1). When mice were treated with a single dose of N6022 at 24 h prior to the MCh challenge, BALF biomarkers were restored toward the levels in the non-sensitized mice (Table 1). For most of these biomarkers, significant efficacy was observed at the lowest N6022 dose of 0.1 mg/kg that was assessed within the panel, while no further benefit was apparent with the higher dose of 1 mg/kg N6022 (Table 1).

**Table 1:** Biomarker patterns in BALF

**Biomarker**^**1**^	**Non-sensitized**	**OVA-sensitized**	**N6022 0.1 mg/kg**	**N6022 1 mg/kg**
**Eotaxin**	2.0 ± 0.5*	35.7 ± 13.8	3.8 ± 0.7*	7.3 ± 1.8*
**Fibrinogen**	2798.0 ± 697.7	52020.0 ± 15925.8	3820.0 ± 453.2*	7120.0 ± 1338.4*
**FGF-9**	606.0 ± 64.0*	1022.0 ± 160.7	566.0 ± 39.2*	598.0 ± 40.8*
**FGF-2**	1830.0 ± 311.3*	4540.0 ± 429.7	2560.0 ± 485.4*	3000.0 ± 320.9*
**KC/GROα**	ND^2^	29.8 ± 8.7	ND	ND
**IFNγ**	2.3 ± 0.3*	10.3 ± 2.5	3.6 ± 0.8*	5.3 ± 0.6*
**IP-10**	2.5 ± 0.2*	15.5 ± 5.3	3.5 ± 0.4*	3.7 ± 0.4*
**IL-1β**	ND	536.0 ± 36.0	ND	ND
**IL-4**	8.4 ± 1.1*	42.8 ± 10.2	10.7 ± 1.0*	14.4 ± 1.6*
**IL-5**	ND	488.2 ± 179.7	ND	ND
**IL-6**	ND	76.3 ± 32.7	ND	ND
**IL-7**	27.8 ± 2.9*	74.6 ± 14.9	22.2 ± 3.0*	33.4 ± 1.7*
**IL-10**	65.6 ± 3.1*	110.6 ± 16.7	60.4 ± 8.2*	87.2 ± 8.3
**IL-11**	20.6 ± 0.7*	38.0 ± 9.1	19.0 ± 1.1*	20.2 ± 1.0*
**IL-12p70**	22.2 ± 3.5*	48.4 ± 8.2	23.6 ± 3.9*	32.4 ± 5.2
**IL-17A**	1.1 ± 0.1*	2.4 ± 0.4	1.6 ± 0.3	1.7 ± 0.2
**IL-18**	642.2 ± 72.0*	7180.0 ± 1438.2	1252.0 ± 221.1*	1152.2 ± 220.1*
**Lymphotactin**	7.1 ± 1.1*	21.4 ± 4.2	10.7 ± 1.2*	11.0 ± 1.3*
**MDC**	11.6 ± 1.9*	203.0 ± 68.5	24.2 ± 5.6*	22.2 ± 4.2*
**MIP-1α**	ND	1798.0 ± 321.1	404.0 ± 72.8*	378.0 ± 55.2*
**MIP-1β**	31.8 ± 2.7*	838.4 ± 158.9	36.6 ± 3.2*	49.4 ± 3.6*
**MIP-1γ**	109.2 ± 27.6*	2024.0 ± 625.3	250.0 ± 46.3*	182.0 ± 32.9*
**MIP-2**	4.3 ± 0.8*	39.8 ± 6.5	5.2 ± 0.8*	7.4 ± 1.5*
**MIP-3β**	55.2 ± 12.2*	315.4 ± 69.1	113.4 ± 21.7*	129.2 ± 27.3*
**MMP-9**	180.6 ± 38.7*	27180.0 ± 11205.5	230.0 ± 24.3*	308.0 ± 60.8*
**MCP-1**	ND	87.2 ± 31.3	ND	ND
**MCP-3**	1.6 ± 0.1*	142.0 ± 36.0	3.0 ± 0.6*	2.8 ± 0.5*
**MCP-5**	0.4 ± 0.1*	3.6 ± 0.9	0.7 ± 0.2*	0.7 ± 0.2*
**MPO**	1550.0 ± 284.6*	435600.0 ± 104051.2	4500.0 ± 545.0*	7580.0 ± 1211.0*
**SAP**	43.4 ± 1.7*	144.8 ± 35.6	49.0 ± 4.6*	52.0 ± 2.3*
**SCF**	77.2 ± 6.6*	288.0 ± 67.9	80.0 ± 9.4*	97.0 ± 9.3*
**RANTES**	0.0019 ± 0.0004*	0.0077 ± 0.0025	0.0025 ± 0.0004*	0.0027 ± 0.0003*
**TIMP-1**	23.6 ± 2.8*	5476.0 ± 2394.1	34.0 ± 5.2*	30.2 ± 2.4*
**TNFα**	11.5 ± 2.9*	42.0 ± 10.0	11.1 ± 1.3*	14.7 ± 2.1*
**sVCAM-1**	522.0 ± 99.1*	4328.0 ± 1669.3	898.0 ± 127.8*	736.0 ± 106.1*

BALF was collected from mice by lavaging both lungs with one mL PBS four times. Biomarker levels were determined using a multi-analyte profile of biomarker patterns. Values are the means ± SEM (N = 5).

*p < 0.05 vs. OVA-sensitized, One-way ANOVA, Dunnett’s.

^1^Reported in pg/mL except for fibrinogen and SAP which are reported in ng/mL. ^2^ND = not detectable.

Biomarker patterns also were evaluated in plasma to determine systemic effects in the model. Although there were substantially fewer systemic biomarkers affected in comparison to BALF, significant changes in asthma-specific biomarkers were noted (Table 2). These changes included significant elevations in fibrinogen, haptoglobin, and interleukins and a significant lowering of immunoglobulin A (IgA) in the plasma from OVA-sensitized mice compared to non-sensitized mice (Table 2). As observed in the BALF, treatment with N6022 significantly restored levels of these plasma markers toward baseline values although there appeared to be slightly greater efficacy achieved with the higher dose of 1 mg/kg N6022 (Table 2).

**Table 2:** Biomarker patterns in plasma

**Biomarker**	**Non-sensitized**	**OVA-sensitized**	**N6022 0.1 mg/kg**	**N6022 1 mg/kg**
**Fibrinogen**	74540.0 ± 5605.7*	126250.0 ± 11070.8	32240.0 ± 7486.6*	68560.0 ± 4251.7*
**Haptoglobin**	22.0 ±4.3*	103.8 ±11.6*	43.4 ±13.8*	33.8 ±8.4*
**IgA**	366.2 ± 14.6*	236.8 ±10.0	347.2 ±14.8*	344.2 ±11.3*
**IL-5**	1.1 ±0.1*	2.3 ±0.3*	1.2 ±0.2*	1.0 ± 0.1*
**IL-18**	45.4 ±1.6*	63.0 ±2.1	51.4 ±6.5	50.6 ±2.4*

Plasma biomarker levels were determined using a multi-analyte profile of biomarker patterns. Values are the means ± SEM (N = 5) in either μg/mL (Fibrinogen, Haptoglobin, and IgA) or ng/mL (IL-5 and IL-18).

*p < 0.05 vs. OVA-sensitized, One-way ANOVA, Dunnett’s.

### NFκB activity in the lung

**Figure 2:**
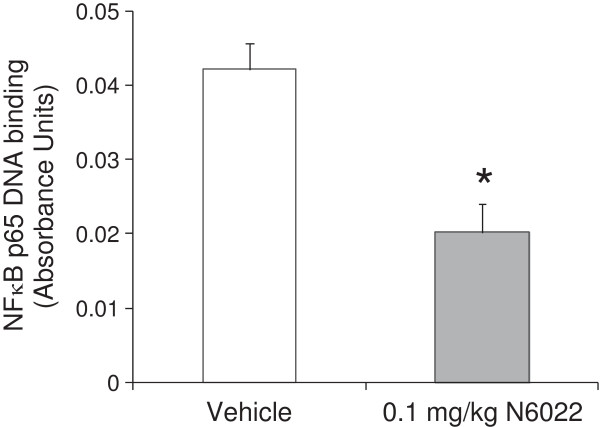
**N6022 decreased NFκB activity in the lungs.** OVA-sensitized mice were treated with a single i.v. dose of either N6022 or PBS vehicle administered 24 h prior to MCh challenge which was one hour prior to the last OVA airway challenge. Nuclear proteins were extracted from lung homogenates and tested for their ability to bind to the DNA consensus sequence for NFκB DNA via immunoassay as a measure of NFκB function. Bars are the means ± SEM of 5 mice per group. *p < 0.05 for N6022 vs. vehicle, two-tailed Student’s t-test.

The effect of N6022 treatment on NFκB function was determined due to the important role of this transcription factor as an upstream regulator for inflammation and tissue repair events associated with asthma [36]. In addition, NO has been shown to modulate NFκB function and activity [37,38]. *In vivo* administration of N6022 in the OVA mouse model led to a significant decrease in active, nuclear NFκB p65 subunit within mouse lung tissue when compared to vehicle (Figure 2). Thus, GSNOR inhibition by N6022 results in the down-regulation of NFκB activation *in vivo*.

### NO and inflammatory dependent mechanisms

**Figure 3:**
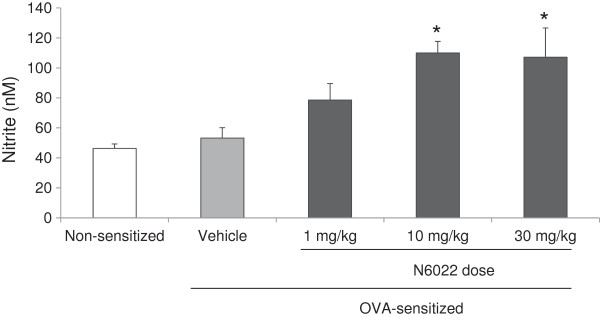
**N6022 increased BALF nitrite.** OVA-sensitized mice were treated with a single i.v. dose of either N6022 or PBS vehicle administered 24 h prior to MCh challenge which was one hour prior to the last OVA airway challenge. Non-sensitized mice treated with a single i.v. dose of PBS at 24 h prior to MCh challenge (one hour prior to OVA) also were assessed. Nitrite levels in BALF were measured via ozone chemiluminescence. Bars are the means ± SEM of 6 mice per group. *p < 0.05 for N6022 vs. vehicle, One-way ANOVA, Dunnett’s.

**Figure 4:**
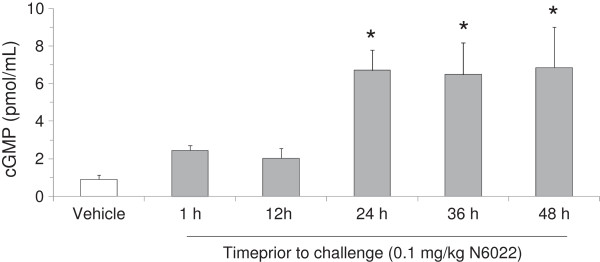
**N6022 treatment increased plasma cGMP.** OVA-sensitized mice were treated with a single i.v. dose of N6022 administered 1 h to 24 h prior to MCh challenge. PBS vehicle was given as a single i.v. dose 24 h prior to MCh challenge. Dosing at 24 h prior to MCh challenge occurred one hour prior to the last OVA challenge on study day 22. Plasma cGMP was assessed via enzyme immunoassay. Bars are the means ± SEM of 5 mice per group. *p < 0.05 for treatment vs. vehicle, One-way ANOVA, Dunnett’s.

The ability of GSNOR inhibition to modulate NO levels and function was determined by measuring BALF nitrite and plasma cGMP in samples from the N6022 mouse OVA studies. Nitrite was measured as one of the stable end products of NO [39], while cGMP was utilized as a marker of NO mediated activity on smooth muscle relaxation [36]. N6022 caused a dose-dependent increase in nitrite, with significant elevation compared to vehicle control at N6022 doses ≥ 10 mg/kg (Figure 3). At N6022 doses < 1 mg/kg, nitrite levels were not increased over vehicle control (data not shown). BALF nitrate levels in PBS vehicle treated non-sensitized mice were similar to vehicle treated OVA sensitized mice (Figure 3). BALF nitrate levels also were determined and were found not to differ among test groups (data not shown). Low molecular weight SNO levels in BALF were below the limits of detection (data not shown). N6022 treatment increased plasma cGMP, with significant elevations over vehicle control when dosed from 24 h to 48 h prior to the MCh challenge (Figure 4).

### Direct actions on airway smooth muscle tone

**Figure 5:**
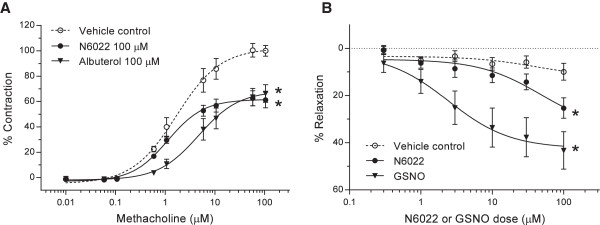
**N6022 attenuated MCh-induced contraction in isolated tracheal rings.** Rat tracheal rings were mounted in a small vessel wire myograph and pretreated with PBS vehicle, N6022, or albuterol for 30 min, after which cumulative doses of MCh were added to induce contraction **(A)**. In other tests, tracheal rings were contracted with 1 μM MCh (EC_40_) followed by cumulative dose additions of N6022 or GSNO to induce relaxation **(B)**. Values are the means ± SEM of 6 to 8 tracheal rings per treatment for **(A)** and 4 to 16 tracheal rings per treatment for **(B)**. *p < 0.05 N6022 vs. vehicle at MCh doses ≥ 5 μM and *p < 0.05 Albuterol vs. vehicle at MCh doses ≥ 1 μM, Two-way ANOVA, Bonferroni’s for **(A)**. *p < 0.05 N6022 vs. vehicle at N6022 doses of 100 μM and *p < 0.05 GSNO vs. vehicle at GSNO doses≥≥ 3 μM, Two-way ANOVA, Bonferroni’s for **(B)**.

The ability of N6022 to directly affect smooth muscle tone in the airways was determined using tracheal ring assays. In rat tracheal rings, pretreatment of the rings with 100 μM N6022 for 30 min caused a significant attenuation of airway smooth muscle contraction induced by cumulative doses of MCh (Figure 5). Significant effects of N6022 were evident at MCh doses ≥ 5 μM when compared to PBS vehicle treated rings. Albuterol was tested as a control and also showed the expected attenuation of tracheal smooth muscle contraction induced by MCh under the same experimental conditions (Figure 5A).

The ability of N6022 to relax tracheal rings following MCh contraction also was determined. In these tests, N6022 demonstrated a dose-dependent relaxation with significant effects at 100 μM compared to rings treated with equivalent volumes of PBS vehicle (Figure 5B). GSNO was tested as a control and showed a dose-dependent relaxation with significant effects at GSNO doses ≥ 3 μM compared to rings treated with equivalent volumes of PBS vehicle (Figure 5B).

### N6022 onset and duration of action

**Figure 6:**
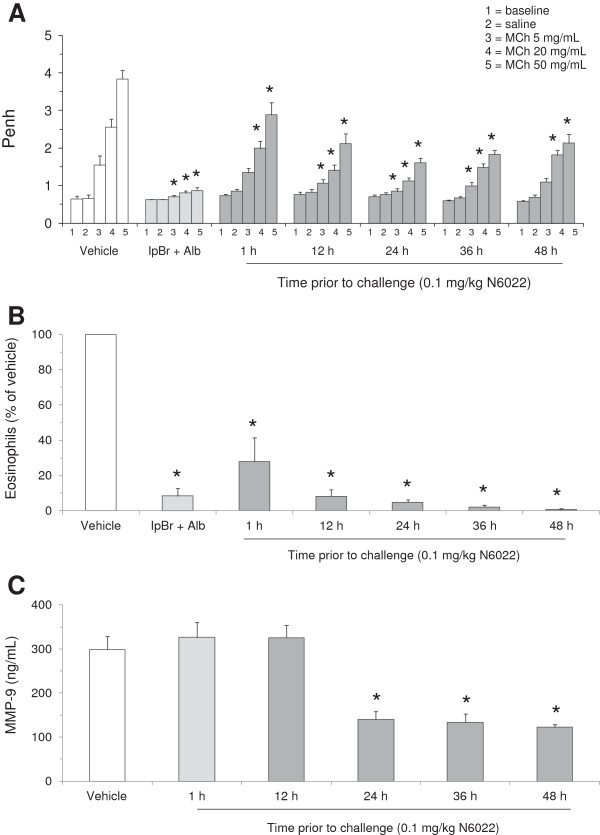
**N6022 exhibited a sustained duration of action in OVA-sensitized mice.** OVA-sensitized mice were treated with a single i.v. dose of N6022 administered 1 h to 48 h prior to MCh challenge. PBS vehicle was given as a single i.v. dose 24 h prior to MCh challenge. IpBr + Alb was administered as inhaled doses at 48 h, 24 h, and 1 h prior to MCh as a positive control for the model. Dosing at 24 h prior to MCh challenge occurred one hour prior to the last OVA challenge on study day 22. Penh was measured at baseline and after challenge with saline followed by increasing doses of MCh aerosol. BALF eosinophils were quantified via light microscopy. Plasma MMP-9 was measured via ELISA. N6022 attenuated Penh **(A)**, BALF eosinophils **(B)**, and plasma MMP-9 **(C)** in OVA-sensitized mice starting as early as one hour with effects sustained for up to 48 h. Bars are the means ± SEM of 10 mice per group for **(A)** and **(B)**, and 5 mice per group for **(C)**. *p < 0.05 for treatment vs. vehicle, One-way ANOVA, Dunnett’s.

**Figure 7:**
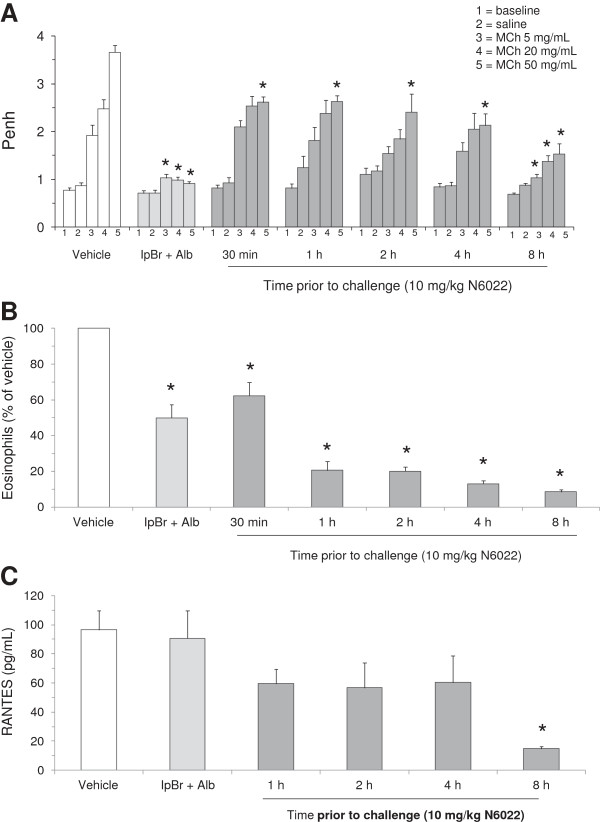
**N6022 exerted a rapid onset of action in OVA-sensitized mice.** OVA-sensitized mice were treated with a single i.v. dose of N6022 administered 30 min to 8 h prior to MCh challenge. PBS vehicle was given as a single i.v. dose 8 h prior to MCh challenge. IpBr + Alb was administered as inhaled doses at 48 h, 24 h, and 1 h prior to MCh as a positive control for the model. Penh was measured at baseline and after challenge with saline followed by increasing doses of MCh aerosol. BALF eosinophils were quantified via light microscopy. Plasma RANTES was measured via enzyme immunoassay. N6022 attenuated Penh **(A)**, BALF eosinophils **(B)**, and plasma RANTES **(C)** in OVA-sensitized mice starting as early as 30 minutes with more pronounced effects occurring at 8 h. Bars are the means ± SEM of 10 mice per group for **(A)** and **(B)**, and 4 mice per group for **(C)**. *p < 0.05 for treatment vs. vehicle, One-way ANOVA, Dunnett’s.

Studies assessing the time course of N6022 effect in the mouse OVA model were performed to explore the onset and duration of action of this compound (Figures 6 and 7). Administration of a single i.v. dose of 0.1 mg/kg N6022 at 1 h to 48 h prior to MCh challenge caused significant decreases in Penh upon MCh exposure at all time points assessed in this study in comparison to vehicle treated mice (Figure 6A). N6022 also significantly decreased BALF eosinophils at all time points in comparison to vehicle treated mice (Figure 6B). There appeared to be a time-dependent influence of N6022 on both Penh and eosinophilia, with greater efficacy observed from 12 h to 48 h. The actions of N6022 were comparable to those observed for the positive control (three IH doses of IpBr + Alb) which caused significant decreases in Penh at 5, 20, and 50 mg/mL MCh challenges (Figure 6A) and a significant lowering of eosinophils (Figure 6B).

In a second time course study, the effect of N6022 administered at 30 min to 8 h prior to MCh challenge was assessed to more fully explore the onset of action. N6022 caused significant decreases in Penh at 50 mg/mL MCh challenge when dosed at 10 mg/kg i.v. at 30 min to 4 h prior to MCh challenge in comparison to vehicle treated mice (Figure 7A). When given at 8 h prior to MCh challenge, N6022 caused significant decreases in Penh at all doses of MCh challenge (Figure 7A). In this study, N6022 also significantly lowered the number of eosinophils compared to vehicle (Figure 7B). As observed above in the onset and duration of action study, the effects of N6022 were dependent on time of N6022 dosing prior to the MCh challenge, with greater benefit observed at the latest time point (8 h) assessed. The three IH doses of the positive control, IpBr + Alb, showed the expected significant effects on Penh and BALF eosinophils (Figures 7A and 7B).

Two biomarkers involved in inflammatory and tissue repair events in asthma were measured in these time course studies to further explore the time-dependent anti-inflammatory influences of N6022 in the OVA asthma model. As shown in Figure 6C, N6022 significantly decreased plasma levels of MMP-9, an inflammatory biomarker and chemokine involved in tissue turnover and repair [40]. N6022 treatment lowered MMP-9 compared to vehicle controls starting after 12 h, with significant and similar actions achieved when administered from 24 h to 48 h prior to MCh challenge (Figure 6C). N6022 also attenuated plasma levels of RANTES (Figure 7C), a cytokine responsible for eosinophil recruitment [41]. N6022 treatment lowered plasma RANTES compared to vehicle controls when administered from 1 h to 4 h prior to MCh, with significant and maximal action achieved when administered at 8 h prior to MCh in this study (Figure 7C). Treatment of mice with three inhaled doses of IpBr + albuterol did not affect RANTES levels compared to vehicle (Figure 7C).

## Discussion

Results of these studies show that N6022, a potent and selective inhibitor of GSNOR activity, has significant bronchodilatory and anti-inflammatory effects in a mouse model of allergic asthma. The effects of N6022 occurred as early as 30 min post-administration, were greater at 12 h and later after administration, and were sustained for at least 48 h after administration. Efficacy with N6022 was achieved with a single i.v. dose and was comparable to that observed after administration of three inhaled doses of the anti-cholinergic, ipratropium bromide, combined with the β2 agonist, albuterol.

N6022 also lowered Penh following MCh exposure in non-sensitized mice and decreased MCh-induced smooth muscle contraction in the tracheal ring assays. These findings show that GSNOR inhibition by N6022 directly influences smooth muscle tone in the airways. However, the potency of N6022 on Penh was approximately 100-fold greater in OVA-sensitized mice compared to non-sensitized mice as there were significant and similar actions at 0.01 mg/kg vs. 1 mg/kg N6022 in OVA- vs. non-sensitized mice, respectively. This difference suggests an important contribution of anti-inflammatory mechanisms on mitigating AHR in response to MCh challenge.

In fact, considerable anti-inflammatory actions of GSNOR inhibition by N6022 were evident as noted by significant reductions in BALF eosinophils as well as BALF and systemic inflammatory biomarkers explored in both the dose response and time course OVA studies. These potent anti-inflammatory actions of N6022 may occur in part through NFκB pathways. NFκB has an important role as an upstream regulator of inflammatory signals, including signals in asthma and the asthma-associated biomarkers that were measured in the current study [37]. Additionally, NFκB is regulated in part by nitrosation of key cysteine residues which leads to a decrease in NFκB function [37,38]. Our data demonstrating the ability of N6022 to decrease NFκB DNA binding in lungs from the OVA mouse studies suggest that GSNOR inhibition likely attenuates inflammation at least in part by down-regulating NFκB activation. Given that N6022 treatment also increased BALF nitrite and plasma cGMP, endpoints used as markers of bioavailable NO [42,43], the anti-inflammatory effects of GSNOR inhibition are consistent with SNO-dependent inhibition of NFκB-mediated signaling. Previously published studies suggest that this SNO-mediated effect may occur through inhibition of transcription factor DNA binding activity [44] or inhibition of pathway activation via nitrosation of IKKβ [45].

The difference in potency observed for N6022 in OVA-sensitized compared to non-sensitized mice also may be explained by differences in activities of the restored GSNO and SNO pools and down-stream nitrosation targets. For example, restoring the levels of GSNO/SNOs may mitigate against disease, whereas in non-disease states, these levels are sufficient and no further benefit or effect is achieved or measurable upon GSNOR inhibitor treatment. In support of this hypothesis, treatment of rats with a related GSNOR inhibitor decreases blood pressure and nitric-oxide dependent flow mediated vasodilation in a salt-induced hypertensive rat model, whereas no effect of the GSNOR inhibitor is noted in normotensive rats [46].

The bronchodilatory capacity observed with N6022 administration is consistent with observations reported in GSNOR knock-out mice, which showed that genetic deletion of GSNOR protected mice from MCh-induced bronchoconstriction compared to wild-type control mice [18]. Similar to N6022, more pronounced effects were evident in GSNOR knock-out mice under the conditions of OVA-induced asthma compared to the non-sensitized model. However, in contrast to the potent anti-inflammatory actions of N6022 in the mouse OVA model, inflammatory responses (BALF eosinophils, BALF IL-13, and serum IgE) were not decreased in GSNOR knock-out mice upon exposure to OVA. The reason for differences in anti-inflammatory influences between genetic deletion and pharmacologic inhibition is not clear, but may be due to differences between life-long homozygous gene deletion of GSNOR and the reversible inhibition of GSNOR activity with a pharmacologic approach. Further contributing factors may include differences in the experimental model such as mouse strain, asthma induction protocol, and endpoints measured.

NO produced from iNOS, upon up-regulation of this enzyme during inflammation, is known to be increased in the expired breath of asthmatics [21], and plays a significant role in the inflammatory responses observed in atopic asthma [19,21,47]. Thus, increasing the pool of bioavailable NO through GSNOR inhibition may appear contradictory. However, there are lowered concentrations of SNOs in the lungs of asthmatic patients [13] even in the presence of the increased exhaled NO [19,20,48] which may be explained by increased GSNOR activity. These findings suggest that the mechanisms by which SNO pools mediate bronchodilatory and anti-inflammatory effects are distinct from the actions of the relatively high concentrations of NO produced by iNOS. These findings also suggest that iNOS derived NO is not necessarily responsible for SNO levels observed in the BALF. In support of this hypothesis, GSNOR inhibitors attenuate iNOS protein expression in cellular models of cytokine-stimulated inflammation ( [49] and SCM, GJR unpublished results). Our studies suggest that inhibition of GSNOR, and the likely subsequent elevation of SNOs, result in attenuation of proinflammatory mediators, in part via down regulation of NFκB signaling. Inhibition of GSNOR as a mechanism to increase SNO pools is thus plausible and of potential benefit in asthma therapy, as noted by efficacy in the mouse model of asthma in the current studies. These proposed mechanisms are consistent with data demonstrating protection from experimental asthma in the GSNOR knock-out mouse [18] and the attenuation of asthma severity in an OVA model following GSNO administration [50]. Taken together, decreased local levels of SNOs through increased GSNOR activity in asthma patients may be an important component of asthma pathophysiology as previously suggested [13,14].

The results and mechanisms noted in the current studies are consistent with other observations in inflammatory disease models in which dysregulated GSNOR and/or altered SNO homeostasis may have important roles. In particular, the pathophysiology of diseases of the respiratory [12,13,20], gastrointestinal [29,30], and cardiovascular [27,28] systems involve inflammatory and NO-mediated pathways which have the potential to be regulated by GSNOR. N6022 and other inhibitors of GSNOR have been shown to decrease inflammation and disease severity in animal models of tobacco smoke induced chronic obstructive pulmonary disease [51], chemically induced colitis [52], acetaminophen induced hepatotoxicity [53], and high salt diet induced hypertension [46].

Direct measurements of airway mechanics were not performed in the current studies, but rather Penh was derived via whole body plethysmography with a Buxco chamber and used as an index of AHR. This technique was chosen as it offers a noninvasive method to measure lung mechanics in unanesthetized and unrestrained mice while allowing for MCh challenge via aerosol/inhalation exposure. While some controversy exists as to the adequacy of Penh as a measure of AHR [54,55], Penh has been shown to be a valid measure of AHR in allergen sensitized mice and to positively correlate with a direct measure of airway resistance using mechanical ventilation in anesthetized and surgically implemented mice of the same strain utilized in these studies [33].

There were some questions that could not be addressed in these studies due to analytical limitations. Although N6022 is a potent and selective inhibitor of human GSNOR activity *in vitro*[31,32], inhibition occurs via a reversible process which precludes the direct measurement of GSNOR inhibition *in vivo* since tissue processing and dilution leads to dissociation of GSNOR inhibitors from the enzyme-substrate complex [31]. Another limitation was the inability to detect GSNO and SNOs in mouse lung or BALF samples. SNOs were assessed using ozone chemiluminescence detection with a nitric oxide analyzer (Sievers) following tri-iodide reduction after prior treatment with sulfanilamide to remove contaminating nitrite signal [56,57]. The detection limit of this assay was 5 pmoles or 50 nM.

GSNOR inhibition in these studies may have indeed caused increased GSNO as suggested by the effects on endpoints influenced by GSNO including bronchodilation, increased BALF nitrite, increased plasma cGMP, and decreased NFκB activity. Because GSNOR can catalyze the reduction of certain aldehydes in addition to the oxidation of GSNO [10,11], an alternative consideration is that the physiological effects of GSNOR inhibition could be due to inhibition of aldehyde reduction rather than the GSNO oxidation reaction. However, there is no evidence that the aldehyde substrates are involved in the endpoints mentioned above, whereas GSNO has been shown in many studies to influence these measurements [13,58,59].

Direct measurement of endogenous GSNO and SNOs is challenging because levels are usually below the limits of detection of current methods. Other investigators also state the inability to detect GSNO in the BALF of asthma patients [13,60]. In this example, the investigators measured high and low molecular weight (i.e., GSNO) SNOs using photolysis-chemiluminescence in the absence or presence of HgCl_2_ to cleave thiol-bound NO. The limit of detection was 2 pmoles. Values reported for total SNOs were 10–20 pmoles/mL (10–20 nM) which are close to our detection limit of 50 nM. It was noted that N6022 did increase BALF nitrite which was utilized as a stable marker of NO, although the detection of nitrite did not correlate with N6022 efficacy at every dose. Similar disparities between physiologic or pharmacologic effects and GSNO levels have been noted in a study showing that efficacy of GSNO was evident at lower doses than those that caused increased BALF SNOs in experimental asthma [49].

## Conclusions

GSNOR inhibition with N6022 in an experimental model of asthma demonstrated significant efficacy toward key parameters associated with asthma including AHR in response to MCh challenge, pulmonary eosinophilia, and both pulmonary and systemic inflammatory biomarkers. GSNOR has recently emerged as a potential target in human asthma and other inflammatory lung diseases. The role of GSNOR in human disease, along with the current findings with the GSNOR inhibitor, N6022, point to GSNOR inhibition as a novel target for the treatment of asthma and other inflammatory lung disease. This rationale has prompted the current evaluation of N6022 in clinical trials for the treatment of inflammatory lung disease including asthma and cystic fibrosis.

## Abbreviations

AHR: Airway hyper-responsiveness; Alb: Albuterol; ANOVA: Analysis of variance; BALF: Bronchoalveolar lavage fluid; CCL-5: Chemokine ligand 5; EC40: Effective concentration 40%; ED50: Effective dose 50%; FGF: Fibroblast growth factor; GSNO: S-nitrosoglutathione; GSNOR: S-nitrosoglutathione reductase; IFNγ: Interferon gamma; IgA: Immunoglobulin A; IH: Inhaled; IL: Interleukin; IL-12p70: Interleukin-12 subunit p70; iNOS: Inducible nitric oxide synthase; IP-10: Interferon gamma induced protein 10; i.p.: Intraperitoneal; IpBr: Ipratropium bromide; i.v.: Intravenous; KC/GROα: Keratinocyte chemoattractant/growth regulated oncogene alpha; MCh: Methacholine; h: Hour or hours; min: Minute or minutes; MCP: Monocyte chemotactic protein; MDC: Macrophage derived chemokine; MIP: Macrophage inflammatory protein; MMP-9: Matrix metalloproteinase 9; MPO: Myeloperoxidase; NFκB: Nuclear factor kappa B; NO: Nitric oxide; NOS: Nitric oxide synthase; OVA: Ovalbumin; PBS: Phosphate buffered saline; Penh: Enhanced pause; SAP: Serum amyloid P-component; RANTES: Regulated upon activation normal T-cell expressed and secreted; sVCAM-1: Soluble vascular cell adhesion molecule-1; TIMP-1: Tissue inhibitor of metalloproteinases-1; TNFα: Tumor necrosis factor alpha.

## Competing interests

All authors are employed or were previously employed (LHG, JPR, and GJR) by N30 Pharmaceuticals, Inc. and hold stocks and/or shares in this company. XS, JQ, and GJR are authors on patents pertaining to content contained in this manuscript. N30 Pharmaceuticals is financing the preparation and processing charges for this manuscript. None of the authors will gain or lose financially or non-financially upon publication of this manuscript.

## Authors’ contributions

JPB participated in design and coordination of the study, analysis and interpretation of data, statistical analyses, and drafted the manuscript. SCM participated in acquisition, analysis, and interpretation of data, statistical analyses, and helped draft the manuscript. XS and JQ participated in N6022 synthesis and critical review of the manuscript. LG, NKM, and RB carried out immunoassays and NFκB assays. MS and KL carried out tracheal ring bioassays. CD carried out nitrite determinations. JPR and DL participated in analysis and interpretation of data and critical review of the manuscript. CS participated in study concept and design and interpretation of data. GJR participated in study concept and design, interpretation of data, and critical review of the manuscript. All authors read and approved the final manuscript.

## Pre-publication history

The pre-publication history for this paper can be accessed here:

http://www.biomedcentral.com/1471-2466/14/3/prepub
